# Burnout and engagement profiles of emergency nurses: the role of job insecurity appraisal and capabilities

**DOI:** 10.3389/fpsyg.2025.1504483

**Published:** 2025-10-17

**Authors:** Neil B. Barnard

**Affiliations:** Optentia Research Unit, North-West University, Vanderbijlpark Campus, Vanderbijlpark, South Africa

**Keywords:** emergency nurses, burnout, engagement, capabilities, job insecurity appraisal, sustainable employability model, structural equation modeling, latent profile analysis

## Abstract

Emergency nurses are particularly vulnerable to burnout (a state of extreme tiredness, reduced ability to regulate cognitive and emotional processes, and mental distancing) with far-reaching consequences for individuals, healthcare systems, and society. Working in high-pressure environments marked by traumatic events, intense workloads, irregular shifts, and emotionally charged encounters, emergency nurses must sustain their performance and well-being amid growing job insecurity. This study examined the roles of work capabilities (enabled and achieved work values), burnout, work engagement, and job insecurity appraisals (as either a hindrance or a challenge) in shaping emergency nurses’ sustainable employability. A quantitative, cross-sectional survey design was used, and data were collected via convenience sampling from 204 emergency nurses across 13 hospitals in South Africa. Structural equation modeling, latent profile analysis, and Bolck-Croon-Hagenaars analysis were employed to examine associations, subpopulations, and profile differences. Findings indicated that appraising job insecurity as a challenge positively affected emergency nurses’ capabilities and engagement, while hindrance appraisals were associated with elevated levels of mental distance, cognitive impairment, and emotional impairment. Capabilities were negatively associated with exhaustion and mental distance, and positively associated with engagement. Latent profile analysis identified four distinct burnout and engagement profiles: moderately burned-out (35%), slightly disengaged (38%), healthy engaged (15%), and burned-out (12%). Emergency nurses in the moderately burned-out profile reported significantly lower challenge appraisals than those in the slightly disengaged group. Additionally, the burned-out and moderately burned-out groups reported lower capability scores than the slightly disengaged and healthy engaged profiles, with the healthy engaged group reporting the highest scores overall. These findings underscore the importance of interventions that build work capabilities and support adaptive interpretations of job insecurity. Such efforts are critical for reducing burnout, enhancing engagement, and promoting the sustainable employability of emergency nurses.

## 1 Introduction

The modern world of work is increasingly characterized by precariousness, with job insecurity becoming a persistent and growing concern (Blustein and Flores, 2023). Emergency nurses are among the most affected, facing complex and demanding roles that expose them to traumatic incidents, heavy workloads, irregular shifts, and emotionally charged encounters with patients and their families. These challenging working conditions place emergency nurses at significant risk of burnout, a state that not only threatens their personal well-being (e.g., depression) but also undermines organizational stability through increased turnover intentions and diminished productivity, ultimately straining the broader healthcare system (De Beer et al., 2022). Given the essential role emergency nurses play in delivering urgent, life-saving care, it is crucial to identify mechanisms that can buffer against burnout and enhance work engagement. Drawing on the Sustainable Employability (SE) model (Van der Klink et al., 2016), this study examines how capabilities (valued work outcomes that are enabled by the work environment and achieved by the individual) affect emergency nurses’ burnout and engagement, and how their job insecurity appraisal (as either a challenge or a hindrance) shape these outcomes.

### 1.1 Job insecurity: a world-wide threat

It is well known that the world of work is undergoing rapid and far-reaching transformations. Organizational restructuring, workplace redesign, downsizing, and the increasing integration of intelligent technologies have fundamentally reshaped employment landscapes. These shifts extend beyond individual organizations and employees, with broader societal consequences such as heightened social unrest and declining consumer confidence (Prado-Gascó et al., 2021). The emergence of online labor platforms and the widespread use of flexible or temporary contracts have further disrupted traditional notions of stable employment (De Cuyper et al., 2023). Simultaneously, global economic crises, mounting demands on workers, and worsening work-life balance, exacerbated by reduced staffing and rising workloads, have amplified work-related stress and complexities (Bakker and Demerouti, 2017).

These developments have significantly contributed toward a worldwide rise in precariousness, eroding job security, diminishing benefits, and widening income inequality (McGaughey, 2022). As Standing (2011) noted, the once-prevailing model of lifelong employment under a paternalistic employer has largely disappeared, although these where in any case predominantly only experienced in developed countries. Today, job insecurity has become a defining feature of modern employment worldwide (De Cuyper et al., 2023).

Job insecurity, viewed as the perceived threat to the continuity of one’s employment (De Witte, 1999; Sverke et al., 2002), is now regarded as one of the most pressing psychological risks in the world of work (De Cuyper et al., 2023). It functions as a chronic stressor and structural characteristic of contemporary employment (Scott, 2004; Shoss, 2017), further aggravated by ongoing economic instability, frequent organizational change, and the lasting impact of the COVID-19 pandemic (Langerak et al., 2021).

In the healthcare sector, especially in nursing, job insecurity presents a particular threat. Nurses comprise the largest segment of healthcare professionals and are vital to the delivery of safe, high-quality care (Prado-Gascó et al., 2021). A substantial body of research links job insecurity to negative personal (such as elevated burnout and diminished engagement) and organizational outcomes (such as higher turnover intentions) (Landsbergis et al., 2014; Sora et al., 2010), compromising the ability of healthcare systems to deliver effective care (Prado-Gascó et al., 2021).

However, job insecurity is not experienced uniformly. Personal and contextual resources (such as psychological resilience, social support, and job autonomy) can shape how individuals perceive and respond to it (Li et al., 2020; Mockałło and Widerszal-Bazyl, 2021). A key factor influencing these responses is appraisal: individuals may interpret job insecurity either as a challenge, which could stimulate growth and effort, or as a hindrance, perceived as a threat to well-being and performance (Bazzoli et al., 2021). Hindrance appraisals are typically linked to adverse outcomes, such as emotional exhaustion and lower job satisfaction (Charkhabi, 2019; Sverke et al., 2002). While challenge appraisals can, in some instances, lead to increased motivation and effort (Piccoli et al., 2019). Research findings on the relationship between job insecurity appraisal and functioning at work remain mixed, with evidence of both positive and negative associations (Stynen et al., 2015). Nonetheless, the general consensus is that job insecurity’s harmful consequences on employee well-being, attitudes, and performance are far reaching (Sverke et al., 2002).

Within this precarious global work context characterized by growing job insecurity, emergency nurses serve on the frontlines of complex, high-pressure healthcare environments. Their work involves rapid assessments, triage, critical interventions, and coordination of care across vars departments and specialties (Olorunfemi and Adesunloye, 2024). These actions often carry severe implications, not just for individual patients but for the functionality and resilience of the healthcare system at large (Elder et al., 2020; Alomari et al., 2021; Caulfield et al., 2022).

Beyond job insecurity, emergency nurses are subjected to chronic occupational stressors such as regular exposure to trauma, persistent understaffing, overcrowded emergency rooms, and limited decision-making autonomy (Yang et al., 2024; Power et al., 2022; Adriaenssens et al., 2011). These challenges are compounded by systemic issues such as rigid hierarchies, lack of recognition, and inadequate psychological support (Caulfield et al., 2022). In addition, emergency nurses often confront workplace violence and the emotional toll of treating traumatic injuries and mass casualties (Mani et al., 2024). The convergence of these stressors makes them especially vulnerable to burnout.

### 1.2 Burnout as a global concern among emergency nurses

As emergency nurses navigate increasingly precarious work environments marked by job insecurity and emotional strain, burnout has become an urgent concern. Defined as a work-related state of exhaustion marked by extreme fatigue, a tendency toward mental distancing, and diminished capacity to regulate cognitive and emotional processes (Schaufeli et al., 2020), burnout is a critical occupational phenomenon with far-reaching implications. It has been associated with heightened intentions to leave the job and profession, reduced job satisfaction, increased psychological stress, and symptoms of depression (Huang et al., 2024). Beyond the personal toll, burnout also poses a serious threat to nurse productivity, quality of care, and patient safety (Jun et al., 2021), detrimental to the healthcare offering and, in turn, the larger society.

The study followed the conceptualization by Schaufeli et al. (2020), arguing that burnout comprises four dimensions, namely exhaustion, mental distancing, cognitive impairment, and emotional impairment (Schaufeli et al., 2020; De Beer and Schaufeli, 2024). These dimensions represent the cumulative effects of chronic job stressors that erode nurses’ ability to function effectively and sustainably.

Meta-analytic studies reveal that nearly 30% of emergency nurses experience symptoms of burnout (Gómez-Urquiza et al., 2017). Contributing factors include excessive workloads, limited autonomy, workplace violence, and lack of support (García-Izquierdo and Ríos-Rísquez, 2012; Hetherington et al., 2024; Rozo et al., 2017). The consequences range from emotional exhaustion and anxiety to impaired clinical judgment and poorer patient outcomes (Adriaenssens et al., 2011; Chao et al., 2016; Li et al., 2018).

While burnout has traditionally been viewed as an individual problem, there is increasing recognition of its systemic roots. Emergency departments, known for chronic understaffing, overcrowding, and emotional intensity, illustrate how burnout often arises from organizational conditions (Jun et al., 2021). Preventative strategies are therefore essential to improve both the psychological well-being and working conditions of emergency nurses (Huang et al., 2024).

Despite its severity, burnout often goes undetected until it has escalated. Early identification and the use of validated assessment tools can help mitigate its progression and improve nurses’ well-being and performance (De Beer and Schaufeli, 2024). Yet, to fully understand emergency nurses’ occupational experiences, it is also necessary to examine what contributes to their work engagement.

### 1.3 Engagement as a desired functioning at work

While burnout reflects the depletion of personal resources, work engagement represents their activation. Engagement is a positive, fulfilling, and motivational state of mind, characterized by vigor, dedication, and absorption (Schaufeli et al., 2002). Vigor reflects energy and resilience, dedication involves pride, enthusiasm, and purpose, and absorption denotes deep concentration and immersion in work (Mazzetti et al., 2023).

High work engagement is linked to better emotional well-being, higher job satisfaction, and stronger organizational commitment (Al-Hamdan and Bani Issa, 2022). Engaged emergency nurses are more likely to provide high-quality care, cope under pressure, and enhance patient satisfaction (Keyko et al., 2016). Engagement fosters resilience, creativity, and effective care delivery (Nantsupawat et al., 2024). When supported by good leadership, adequate staffing, and strong team dynamics, emergency nurses are more likely to thrive (Siller et al., 2016; Sawatzky and Enns, 2012; Clark et al., 2020). These outcomes are crucial in emergency settings where both urgency and complexity are ever-present.

Addressing burnout and fostering engagement among emergency nurses in an age of growing job insecurity is therefore not only an organizational and psychological imperative, it is also a societal one. These efforts align with key Sustainable Development Goals (SDGs), including good health and well-being (SDG 3), reducing inequalities (SDG 10), and building strong institutions (SDG 16). Promoting the sustainable employability of emergency nurses is thus central to the future resilience of healthcare systems.

To understand how this can be achieved, this study draws on the Sustainable Employability (SE) model developed by Van der Klink et al. (2016). This theoretical framework brings a normative and social justice lens to the study of work, focusing not only on what individuals do, but on what they are enabled to do and be, based on what they themselves find valuable (Robeyns, 2017). The SE model, with its emphasis on well-being, ethical reasoning, and human dignity, offers a fitting framework for investigating how emergency nurses can sustain optimal functioning in the face of increasing precarity.

## 2 Sustainable employability model as a theoretical framework

Given the vital role emergency nurses play in healthcare systems and the complexity of the environments in which they operate, it is essential to understand how to support their sustained, optimal functioning. The Sustainable Employability (SE) model (Van der Klink et al., 2016), grounded in Amartya Sen’s Capability Approach (CA; Sen, 1985), provides a relevant and practical theoretical lens for examining this issue. Central to the SE model is the idea that well-being and effective functioning at work depend not only on personal effort or individual competencies, but on the real opportunities that the work context affords for people to do and be what they consider important and valuable (Van der Klink, 2019).

Applied to emergency nurses, the SE model suggests that promoting work outcomes such as reduced burnout and enhanced engagement requires understanding what nurses value in their work and whether they are enabled by their work environment to pursue and achieve these valued outcomes. In this regard, the SE model provides a normative framework for understanding how agency and contextual conditions interact to support or constrain sustainable employability (Abma et al., 2016; Fleuren et al., 2020).

The SE model consists of three interrelated components, namely capabilities (the real opportunities to achieve valued work outcomes, shaped by individual values and contextual supports or barriers), functionings (the actual states of being and doing, such as burnout or engagement, that result from realized capabilities), and agency (the freedom to make choices that reflect one’s valued work outcomes at work) (Fleuren et al., 2020; Gürbüz et al., 2024; Van der Klink et al., 2016).

Consequently, this study applies the SE model to examine how emergency nurses’ work capabilities, relate to their burnout and engagement as functionings at work. Furthermore, it investigates how their appraisals (as either a challenge or a hindrance) of job insecurity (as an external constraint) affects their capabilities, burnout, and engagement. The study approach this using both a variable and person-centered perspective to the interplay among these constructs. In doing so, the study aims to provide a nuanced, context-sensitive understanding of emergency nurses’ burnout and engagement, furthering our understanding of their sustainable employability.

To contextualize the study, the key variables (including job insecurity appraisal, capabilities, burnout, and engagement) are discussed in relation to emergency nurses within the Sustainable Employability framework. These variables are discussed in three parts. First, capabilities as the foundation for sustained desired functioning at work. Second, burnout and engagement as functionings at work. Third, job insecurity appraisal as a critical contextual factor affecting emergency nurses’ capabilities and functionings at work.

### 2.1 Work capabilities

Emergency nurses require a broad range of competencies to function in high-pressure, emotionally charged conditions. These include technical skills (such as accurate patient assessment, trauma management, and clinical decision-making) (McCarthy et al., 2013; Trisyani et al., 2023), interpersonal skills (such as communication, collaboration, and leadership) (Komsan et al., 2023), and preparedness for extreme situations (such as handling violent attacks or mass casualty events) (Mani et al., 2024). While these competencies are essential for professional performance (Woo et al., 2017), they reflect only what nurses can do, not what they are actually able to do and be, given the opportunities afforded by their environment (Sen, 1985; Robeyns, 2017). This distinction is crucial. Capabilities encompass not only personal skills, but also the degree to which the work environment enables nurses to act on those skills in ways that align with their values and goals.

Within the SE model, work capabilities are defined as the work outcomes that individuals consider important, are enabled to pursue by their work environment, and are able to achieve in practice (Van der Klink et al., 2016). A value becomes a capability once it is considered important by the worker and achieved through the opportunities, resources, and support available in the work environment.

Philosophically, Sen (1985) advocates a context-sensitive approach to capabilities, resisting fixed lists. In contrast, Nussbaum (2011) proposes a universal set of capabilities to safeguard dignity and justice. This study adopts a combined approach, integrating Sen’s emphasis on contextual flexibility with Nussbaum’s normative clarity by drawing on the work of Abma et al. (2016). Subsequently, this study investigated seven core work values that form the basis of capabilities: (1) using knowledge and skills, (2) developing knowledge and skills, (3) building meaningful relationships at work, (4) earning a good income, (5) contributing to something valuable, (6) involvement in important decisions, and (7) setting personal goals. These values are considered capabilities only when they are considered important by the individual, enabled by the work environment, and achieved by the individual (Abma et al., 2016; Gürbüz et al., 2024).

Research shows that individuals with more comprehensive capability sets report better functioning at work, including lower burnout and higher engagement (Barnard et al., 2023; Murangi et al., 2022; De Wet and Rothmann, 2022). These capabilities tend to cluster in distinct patterns across occupational groups and are affected by external factors such as job demands (Murangi et al., 2022), access to decent work (Ragadu and Rothmann, 2023), and job precariousness (Barnard et al., 2023). This underscores the critical role of supportive organizational contexts in enabling work capabilities and promoting sustainable employability.

Accordingly, this study examines emergency nurses’ capabilities, based on the seven work values proposed by Abma et al. (2016), and investigates how these capabilities are associated with their functioning at work (burnout and engagement), with the aim of deepening our understanding of their sustainable employability.

### 2.2 Work functionings

Functionings, within the SE model, refer to the actual beings (or states) and doings (or actions) of people at work (Van der Klink et al., 2016). Within the SE model, functionings are viewed as direct reflections of a person’s opportunity to pursue and achieve meaningful work outcomes in practice. As highlighted earlier, emergency nurses are especially vulnerable to burnout due to the intense demands of their roles and the increasing global prevalence of job insecurity. It is therefore critical to identify not only how to prevent burnout but also how to promote work engagement as a marker of desired functioning at work.

Importantly, this study does not treat burnout and engagement as opposite ends of a single continuum. Rather, it also adopts a person-centered perspective that recognizes how individuals may experience both states concurrently. For instance, an emergency nurse might feel emotionally exhausted yet still experience a strong sense of meaning and absorption in patient care (Salmela-Aro et al., 2019; Bekker et al., 2024). Acknowledging this coexistence underscores the importance of nuanced, dual-focused interventions, those that both reduce risk and enhance resilience (Bekker et al., 2024; Schaufeli and Bakker, 2004). Moreover, engagement is not merely an outcome. It is supported by access to meaningful work capabilities. When nurses are enabled to pursue personally valued goals (such as autonomy, skill use, social recognition, and contributing to something meaningful) their engagement is more likely to be sustained (Murangi et al., 2022). These capabilities serve as buffers against stress and help nurses remain committed, even in demanding contexts.

To investigate these dynamics, the study takes both a variable-centered approach (examining associations among capabilities, burnout, and engagement) and a person-centered approach (using latent profile analysis [LPA] to identify subgroups of nurses with distinct combinations of burnout and engagement).

The LPA has proven effective in uncovering such profiles across occupational settings. Among researchers, Boone et al. (2022) identified five burnout profiles, including high risk, cynical, and overextended. In the education sector, Salmela-Aro et al. (2019) reported “engaged” and “engaged-burnout” profiles among teachers; and among students, Räihä et al. (2024) found four burnout and engagement profiles. In South African high schools, Bekker et al. (2024) uncovered five profiles related to burnout and engagement (healthy engaged, moderately balanced, slightly disengaged, moderately burned-out, and burned-out). In the healthcare domain, Luna et al. (2023) identified four profiles among medical and nursing personnel, while Leiter and Maslach (2016) reported five among healthcare workers more broadly, each characterized by varying levels of burnout, engagement, workload, and access to resources.

Building on these findings, the study considered it valuable to investigate whether subgroups exist among emergency nurses based on their burnout and engagement, and how these profiles differ in relation to their capabilities and job insecurity appraisal. This dual approach would offer a more granular understanding of how emergency nurses function in the face of complex demands and supports the development of tailored, context-sensitive interventions that promote their sustainable employability.

### 2.3 Job insecurity as an external factor

The SE model emphasizes that contextual factors (both within and beyond the organization) play a central role in shaping people’s capabilities and functioning at work. One particularly relevant external constraint is job insecurity. In line with the SE framework, this study examines job insecurity as a contextual barrier that may affect emergency nurses’ capabilities, burnout, and engagement. Specifically, it investigates how the appraisal (as either a challenge or a hindrance) thereof affects emergency nurses’ capabilities and functioning at work. These appraisals affect motivation, stress responses, and resource availability, particularly in unstable or resource-constrained systems (Bazzoli et al., 2021). For emergency nurses, job insecurity is not just an abstract concern, it intersects with day-to-day experiences of staffing shortages, emotional strain, and unpredictability.

Therefore, this study examines whether emergency nurses’ appraisals of job insecurity affect their capabilities, burnout, and engagement, both individually and across identified profiles. In doing so, it offers a comprehensive, context-sensitive analysis of sustainable employability under conditions of precarity.

## 3 Rationale and aims of the study

Emergency nurses play a vital role in delivering time-sensitive, high-stakes care within healthcare systems. However, their work environments are increasingly defined by uncertainty, limited resources, and escalating demands. These systemic pressures are further intensified by the rising prevalence of job insecurity, a condition that undermines both employee well-being and sustainability.

Despite these risks, some emergency nurses remain engaged, resilient, and committed to their work. This suggests that certain contextual and psychological resources, particularly those that support individuals in pursuing and achieving personally valued work goals, may buffer against burnout and foster engagement.

This study is grounded in the Sustainable Employability (SE) model developed by Van der Klink et al. (2016). The model offers a normative and social justice perspective on how individuals can sustain meaningful and productive working lives, even in the face of external constraints.

Key elements of the SE model include: *work capabilities*, which are the real opportunities individuals have to achieve valued work outcomes; *functionings*, referring to the actual outcomes or states (such as burnout and engagement); and *agency*, comprising the freedom to make choices aligned with personal values and goals. The model also emphasizes the influence of contextual factors (such as job insecurity) in shaping these experiences.

While prior research has linked job insecurity to burnout and lower engagement, little is known about how the appraisal of job insecurity (whether seen as a challenge or a hindrance) interacts with work capabilities in shaping emergency nurses’ work-related experiences. In addition, most studies adopt variable-centered approaches, which can obscure differences between subgroups of emergency nurses who may experience unique combinations of burnout and engagement in relation to capabilities and job insecurity appraisal.

A better understanding of these patterns is essential for designing tailored interventions that address the specific needs and realities of emergency nurses. This study seeks to contribute a more holistic, context-sensitive understanding of emergency nurses’ functioning at work, with implications for policies and practices that support their sustainable employability in increasingly precarious healthcare settings.

Consequently, the study aims to:

1. Examine how emergency nurses’ appraisals of job insecurity (as a challenge or hindrance) relate to burnout and engagement.
2. Investigate the role of work capabilities in shaping these outcomes.
3. Examine whether distinct latent profiles of emergency nurses emerge based on combinations of burnout and engagement.
4. Identify how these profiles differ in terms of job insecurity appraisals and work capabilities.

**Figure 1:**
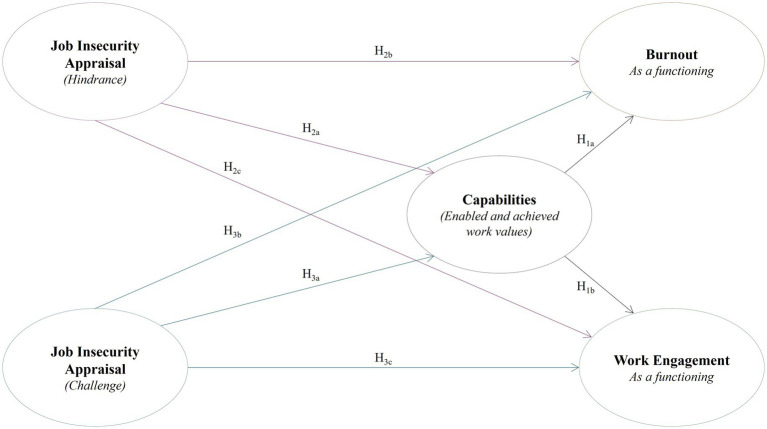
Conceptual model of job insecurity appraisal, capabilities, burnout, and work engagement. H_4_ and H_5_ (not included in figure) concerns the latent burnout and engagement profiles among emergency nurses and its link to job insecurity appraisal and capabilities.

See Figure 1 for the study’s conceptual framework.

### 3.1 Research questions

Guided by the Sustainable Employability (SE) model, this study sought to explore how emergency nurses’ job insecurity appraisals and work capabilities relate to their occupational functioning, specifically, their levels of burnout and engagement. The overarching aim was to examine whether distinct latent burnout and engagement profiles could be identified and how these profiles differ based on job insecurity appraisals and capabilities.

The primary research question of the study is: *“Do emergency nurses exhibit distinct latent burnout and engagement profiles, and how do these profiles differ in relation to their work capabilities and job insecurity appraisals?”*

In support of this research question, the study addressed the following specific research questions:

1. What are the associations among emergency nurses’ job insecurity appraisals (as either a challenge or a hindrance), work capabilities, burnout, and engagement?
2. How many distinct latent burnout and engagement profiles (or subpopulations) can be identified among emergency nurses?
3. In what ways do the identified burnout and engagement profiles differ based on emergency nurses’ job insecurity appraisals?
4. In what ways do the identified burnout and engagement profiles differ based on emergency nurses’ work capabilities?

### 3.2 Hypotheses

Considering previous research and the conceptual foundations of the Sustainable Employability (SE) model, five hypotheses were formulated to guide this study.

In line with the SE model’s central premise, that a comprehensive set of capabilities contributes to desired functionings at work (Fleuren et al., 2020; Gürbüz et al., 2024; Nussbaum, 2011; Sen, 1985), Hypothesis 1 (H_1_) posited that emergency nurses’ work capabilities would be negatively associated with their burnout (H_1a_) and positively with their work engagement (H_1b_).

Within the SE model, contextual factors such as external constraints affects workers’ capabilities and so their functionings at work (Van der Klink et al., 2016). Consequently, Hypothesis 2 (H_2_) was that appraising job insecurity as a hindrance would have a negative effect on emergency nurses’ capabilities (H_2a_) and engagement (H_2c_), and a positive effect on their burnout (H_2b_). Furthermore, it was hypothesized (H_3_) that by appraising job insecurity as a challenge would have a positive impact on emergency nurses’ capabilities (H_3a_) and engagement (H_3c_), and a negative impact on their burnout (H_3b_).

Drawing from previous research that identified latent burnout and engagement profiles among different populations (e.g., Bekker et al., 2024; Boone et al., 2022; Leiter and Maslach, 2016; Luna et al., 2023; Räihä et al., 2024; Salmela-Aro et al., 2019), it was anticipated that emergency nurses would form distinct burnout and engagement profiles based on their scores across the four burnout dimensions and their levels of work engagement. Accordingly, Hypothesis 4 (H_4_) stated that emergency nurses can be grouped into distinct subpopulations (or latent profiles) characterized by different combinations of burnout symptoms and work engagement levels.

Based on prior findings that profile membership often varies by key contextual or psychological factors (e.g., academic boredom, job demands, and resources), it was hypothesized that the identified latent profiles would differ based on emergency nurses’ job insecurity appraisals and work capabilities. Thus, Hypothesis 5 (H_5_) proposed that emergency nurses’ burnout and engagement profile membership will differ based on their work capabilities (H_5a_) and job insecurity appraisals (H_5b_). Specifically, it was anticipated that emergency nurses in profiles marked by high burnout symptoms and low engagement would report lower work capabilities and appraise job insecurity as a hindrance. In contrast, those in profiles with low burnout and high engagement were expected to have higher capabilities and to perceive job insecurity as a challenge.

## 4 Methodology

### 4.1 Research design

A quantitative, cross-sectional survey design was employed, which was well-suited to the study’s aim of examining associations among emergency nurses’ job insecurity appraisals (as a challenge or a hindrance), work capabilities, burnout, and engagement at a single point in time. Cross-sectional designs are widely used in the social sciences to provide a snapshot of variables and their relationships, offering practical advantages such as accessibility, cost-effectiveness, and efficiency in data collection and analysis (Wang and Cheng, 2020).

Although this design does not allow for causal inference or assessment of changes over time, it is valuable for identifying associations among variables and generating insights to guide future research and intervention efforts (Wang and Cheng, 2020). This is particularly relevant given the relatively underexplored nature of the work capabilities construct among emergency nurses in the South African context.

By applying a cross-sectional design, the study provides a foundational understanding of the psychological and contextual dynamics contributing to sustainable employability among emergency nurses. These findings offer important insights for organizational policy, intervention design, and the development of future longitudinal or experimental studies (Spector, 2019).

### 4.2 Participants

Emergency nurses employed by hospitals providing Level 1 or Level 2 trauma care were invited to participate in the study (Trauma Society of South Africa, 2021). Level 1 facilities provide leadership and comprehensive care across the full spectrum of injury (from prevention to rehabilitation) with 24-h access to all primary specialties and a trauma surgeon as director. Level 2 facilities offer initial definitive trauma care for all injury severities, including the core specialties, with 24-h medical coverage.

Both public and private sector hospitals within Gauteng province, South Africa, were approached. The healthcare sector in South Africa comprises both publicly and privately held hospitals. The public sector, funded by the South African government, serves the majority of the population (approximately 71%). In contrast, the private sector is primarily funded through medical aid schemes and private health insurance (Rensburg, 2021).

Thirteen hospitals participated in the study, comprising one public and 12 private facilities drawn from four private hospital groups. Emergency nurses from these hospitals formed the study sample. A total of 204 nurses completed the survey. The sample comprised 72% female participants, who reported an average age of 38 years. Most were permanently employed (75%) and held some form of tertiary qualification: a higher certificate (26%), a three-year diploma (24%), or a bachelor’s degree (21%).

### 4.3 Measuring instruments

The *Job Insecurity-Appraisal Scale* (JIAS; Bazzoli et al., 2021) was used to measure emergency nurses’ job insecurity appraisal. The JIAS uses six items to measure job insecurity primary appraisals, namely, *hindrance* (three items; e.g., “Job insecurity curbs my work efforts”) and *challenge* (three items; e.g., “Job insecurity encourages me to be proficient at my job”). Participants were required to respond to the items using a five-point scale, ranging from 1 (*never*) to 5 (*always*). The JIAS reported acceptable reliability, validity, and strict measurement invariance (Bazzoli et al., 2021).

The *Capability Set for Work Questionnaire* (CSWQ; Abma et al., 2016) was administered to measure emergency nurses’ work capabilities. The CSWQ measures seven work capabilities: (1) use of knowledge and skills; (2) development of knowledge and skills; (3) involvement in important decisions; (4) building and maintaining meaningful contacts at work; (5) setting own goals; (6) having a good income; and (7) contributing to something valuable. The participants were requested to indicate whether (a) they considered the work capability important (*importance*, e.g., “How important is it to you to be able to use your knowledge and skills at work?”), (b) their work was offering them sufficient opportunities to achieve it (*enablement*, e.g., “Does your current work offer you enough opportunities to do that?”), and (c) they succeeded in achieving it (*achievement*, e.g., “To what extent do you succeed in doing so?”). They had to respond to these items using a five-point scale, ranging from 1 (*totally not*) to 5 (*to a very great extent*). The CSWQ is reliable and valid for the South African context (Barnard et al., 2023; Gürbüz et al., 2022; Ragadu and Rothmann, 2023).

The *Burnout Assessment Tool* (BAT; Schaufeli et al., 2020) was distributed to investigate emergency nurses’ burnout. The BAT consists of 23 items measuring four components, namely, *exhaustion* (eight items; e.g., “At work, I feel mentally exhausted”), *mental distance* (five items; e.g., “I struggle to find any enthusiasm for my work”), *cognitive impairment* (five items; e.g., “At work, I have trouble staying focused”), and *emotional impairment* (five items; e.g., “At work, I feel unable to control my emotions”). Participants used a five-point scale, ranging from 1 (*never*) to 5 (*always*) to respond to the items. The BAT reported acceptable reliability and convergent and discriminant validity in previous research (Schaufeli et al., 2020).

Three *Flourishing-at-Work Scale* (FAWS; Rautenbach and Rothmann, 2017) items were used to assess emergency nurses’ work engagement: *physical* (i.e., “During the past month at work, how often did you feel energized when you worked?”), *cognitive* (i.e., “During the past month at work, how often did you focus a great deal of attention on your work?”), and *emotional* (“During the past month at work, how often did you get excited when you performed well on your job?”). Participants responded to the items using a six-point scale, ranging from 1 (*never*) to 6 (*every day*). The Flourishing-at-Work Scale – Short Form (FAWS-SF) reported acceptable results during a validation study conducted in the South African context (Rautenbach and Rothmann, 2017).

### 4.4 Research procedure

Scientific clearance for the study was obtained from the Scientific Committee of the School of Industrial Psychology and Human Resource Management at North-West University. Ethical clearance was granted by the North-West University Health Research Ethics Committee (NWU-HREC) (ethics number: NWU-00273-21-A1). Following ethical approval, permission to conduct the study was obtained from the Provincial Department of Health, the participating public hospital, and the research committees of four private hospital groups. Approval was also secured from hospital management and the heads of the respective emergency departments.

A non-probability sampling approach was used, with emergency nurses selected as the target population due to their relevance to the study’s objectives. A convenience sampling strategy was employed, as Gauteng province provided geographic accessibility to the researcher and hosts a high concentration of healthcare facilities with emergency departments.

Hospitals were approached based on feasibility and willingness to participate. Thirteen hospitals ultimately agreed to take part in the study; 12 from the private sector and one public hospital. While the inclusion of emergency nurses was aligned with the study’s purpose, the overall sampling method reflects convenience, as participation was shaped by access and institutional consent rather than random or systematic selection.

Data were collected through a combination of online and paper-based surveys. An advertisement was distributed to eligible participants via a designated gatekeeper at each hospital. This communication outlined the purpose of the study, participant eligibility criteria, and instructions for participation. The online version included a link to the survey platform (QuestionPro), where participants provided informed consent before accessing the questionnaire.

To enhance participation, the researcher also visited each hospital during shift-change meetings to present the study directly to emergency nurses. During these visits, paper-based survey packets (which included separate informed consent forms) were distributed. The researcher explained the study’s purpose, eligibility criteria, the voluntary nature of participation, and participants’ right to withdraw at any time without consequence. It was emphasized that responses would be treated anonymously and confidentially, and that no identifying information would be collected.

Participants were invited to complete the paper-based survey and return it by placing it into a secure drop-box located in a neutral area of the hospital. Surveys were only retained if informed consent was clearly indicated.

Although a dual-mode data collection strategy was used, the majority of responses (91%) were received through the paper-based method.

### 4.5 Data analysis

The SPSS Version 30 (IBM Corporation, 2020), Jamovi Version 2.3.28 (The Jamovi Project, 2022), and Mplus Version 8.11 (Muthén and Muthén, 2023) statistical software programs were used for the statistical analyses.

To select the model that fitted the data best, numerous goodness-of-fit indices and information criteria were utilized (West et al., 2012): the chi-square statistic (the test of absolute fit of the model), Tucker-Lewis index (TLI), comparative fit index (CFI), standardized root mean residual (SRMR), and root mean square error of approximation (RMSEA). TLI and CFI values higher than 0.90 indicate an acceptable value, with a value higher than 0.95 indicating an excellent fit. Score values for SRMR and RMSEA below 0.08 with a 90% confidence interval, not including 0, indicate acceptable values (Wang and Wang, 2020). The mean- and variance-adjusted weighted least squares (WLSMV) estimator was used to compute the measurement model. Structural equation modeling (SEM) was computed to asses the conceptual model using similar criteria.

McDonald’s omega coefficient (*ω*) was employed to investigate scale reliability, using a 0.70 cut-off value. Spearman correlations were used to assess the associations among emergency nurses’ job insecurity appraisal (hindrance and challenge), work capabilities (enabled and achieved work values), burnout (exhaustion, mental distance, and cognitive and emotional impairment), and work engagement. Point biserial correlations were utilized to compute the relationships with work capabilities.

Latent profile analysis (LPA) was used to identify potential latent emergency nurse burnout and engagement profiles. A model was retained if it significantly improved from the reference model to the model with more specified profiles (or subpopulations). The following criteria were employed to assess and compare the models: Bayesian information criterion (BIC), Akaike information criterion (AIC), and sample-size adjusted Bayesian information criterion (aBIC) values (Kline, 2016; Wang and Wang, 2020). To select the model with the optimal number of profiles, the Lo–Mendell–Rubin test (LMR LR; Lo et al., 2001), the adjusted Lo–Mendell–Rubin test (aLMR), and the bootstrapped likelihood ratio test (BLRT; Wang and Wang, 2020) were used. Entropy values were utilized to assess profile verification. A value close to 1 is indicative of acceptable classification. Average latent profile probabilities were computed to determine the probability of correct profile membership, with a cut-off value above 0.80 being a good indicator (Geiser, 2013).

Bolck-Croon-Hagenaars analysis (BCH; Bolck et al., 2004) compared the identified latent emergency nurse burnout and engagement profiles regarding their job insecurity appraisal and work capabilities.

## 5 Results

### 5.1 Measurement model of job insecurity, work capabilities, burnout, and work engagement

Confirmatory factor analysis (CFA) was used to assess the measurement model of job insecurity appraisal (hindrance and challenge), work capabilities (enabled and achieved work values), burnout (exhaustion, mental distance, cognitive impairment, and emotional impairment), and work engagement. The fit statistics obtained were as follows: *χ*^2^ = 1196.57 (*df* = 674, *p* < 0.001); CFI = 0.95; TLI = 0.95; RMSEA = 0.06 [0.06, 0.07], *p* = 0.001; SRMR = 0.08. The sizes of the factor loadings of the items on their target factors were as follows: job insecurity (hindrance): *λ* = 0.72 to 0.92 (*M* = 0.82); job insecurity (challenge): *λ* = 0.81 to 0.95 (*M* = 0.89); work capabilities: *λ* = 0.73 to 0.85 (M = 0.79); exhaustion: *λ* = 0.71 to 0.88 (*M* = 0.80); mental distance: *λ* = 0.37 to 0.95 (*M* = 0.68); cognitive impairment: *λ* = 0.87 to 0.94 (*M* = 0.90); emotional impairment: *λ* = 0.75 to 0.89 (*M* = 0.82); and work engagement: *λ* = 0.71 to 0.86 (*M* = 0.79). Although one of the exhaustion items (i.e., “At work, I feel mentally exhausted”) reported a low factor loading (*λ* = 0.22), it was decided to keep the item, as the overall fit statistics were acceptable. Consequently, the factors were well defined and aligned with theoretical expectations.

### 5.2 Descriptive statistics, reliabilities, and correlations

Table 1 reports the scale reliabilities, means, standard deviations, and Pearson correlations of the study variables. McDonald’s omega coefficients above 0.70 were obtained for all scales. Therefore, all instruments used in the study to investigate the respective variables showed acceptable reliability (Nunnally and Bernstein, 1994).

**Table 1:** Descriptive statistics, reliabilities, and correlations of the scales.

Variable	ω	Mean	*SD*	(1)	(2)	(3)	(4)	(5)	(6)	(7)
(1) Job insecurity (hindrance)	0.83	2.83	0.96	–	–	–	–	–	–	–
(2) Job insecurity (challenge)	0.89	3.18	1.09	0.40***	–	–	–	–	–	–
(3) Work capabilities	0.81	0.50	0.33	0.16*	0.33***	–	–	–	–	–
(4) Exhaustion	0.79	3.17	0.95	0.06	−0.13	−0.29***	–	–	–	–
(5) Mental distance	0.71	2.76	0.97	0.17*	−0.05	−0.28***	0.83***	–	–	–
(6) Cognitive impairment	0.86	1.74	0.77	0.11	−0.10	−0.09	0.68***	0.77***	–	–
(7) Emotional impairment	0.81	1.78	0.80	0.21**	−0.02	−0.09	0.62***	0.71***	0.86***	–
(8) Work engagement	0.77	4.85	0.99	0.09	0.47***	0.50***	−0.61***	−0.69***	−0.62***	−0.58***

ω = McDonald’s omega coefficient; SD = standard deviation; small effect = *r* ≥ 0.10, medium effect = *r* ≥ 0.30, large effect = *r* ≥ 0.50; ***p < 0.001, **p < 0.01, *p < 0.05.

As seen in Table 1, work capabilities were statistically significantly and positively correlated with the hindrance (small effect) and challenge (medium effect) job insecurity appraisals. Regarding the four burnout dimensions, only the hindrance job insecurity appraisal was statistically significantly related to mental distance and emotional impairment (small, positive effects), while only the challenge job insecurity appraisal was statistically significantly associated with work engagement (medium, positive effect).

Emergency nurses’ work capabilities had a statistically significant, negative association (small effects) with the exhaustion and mental distance burnout components and a positive association with work engagement (large effect). Work engagement was statistically significantly and negatively related to all four burnout components, with large effects.

Not shown in Table 1, the hindrance job insecurity appraisal was statistically significantly associated with the contributing to something valuable work capability (small, positive effect). Meanwhile, besides using knowledge and skills, the challenge job insecurity appraisal variable was positively associated with all seven work capabilities (small effect).

Furthermore, regarding emergency nurses’ burnout, apart from setting their own goals and using knowledge and skills, exhaustion was statistically significantly and negatively related to all work capabilities (small effects). Mental distance was not associated with setting goals and being involved in important decisions. However, it was negatively associated with the other five work capabilities (small effects). Cognitive impairment and emotional impairment were not associated with the seven work capabilities.

Also, emergency nurses’ work engagement was significantly and positively correlated with all seven work capabilities. Contributing to something valuable, earning a good income, and building and maintaining meaningful relationships at work were correlated with medium effects, while the other four work capabilities showed small effects.

### 5.3 Testing the structural model

Structural equation modeling (SEM) was used to test the structural model of job insecurity appraisal (as either a hindrance or a challenge), capabilities, burnout, and work engagement (see Table 2).

**Table 2:** Structural model of job insecurity appraisal, capabilities, burnout, and work engagement.

Independent variable	Dependent variable	*β*	SE	*p*-value
Job insecurity (hindrance)	Capabilities	0.01	0.09	0.897
	Exhaustion	0.13	0.08	0.097
	Mental distance	0.22	0.08	0.006**
	Cognitive impairment	0.17	0.08	0.025*
	Emotional impairment	0.22	0.09	0.009**
	Work engagement	−0.11	0.09	0.231
Job insecurity (challenge)	Capabilities	0.26	0.09	0.005**
	Exhaustion	−0.10	0.09	0.273
	Mental distance	0.01	0.09	0.886
	Cognitive impairment	−0.15	0.08	0.057
	Emotional impairment	−0.09	0.09	0.280
	Work engagement	0.36	0.09	0.000***
Capabilities	Exhaustion	−0.25	0.08	0.001**
	Mental distance	−0.28	0.07	0.000***
	Cognitive impairment	−0.04	0.08	0.600
	Emotional impairment	−0.08	0.09	0.353
	Work engagement	0.34	0.07	0.000***

β = standardized regression coefficient; SE = standard error; ***p < 0.001, **p < 0.01, *p < 0.05.

**Figure 2:**
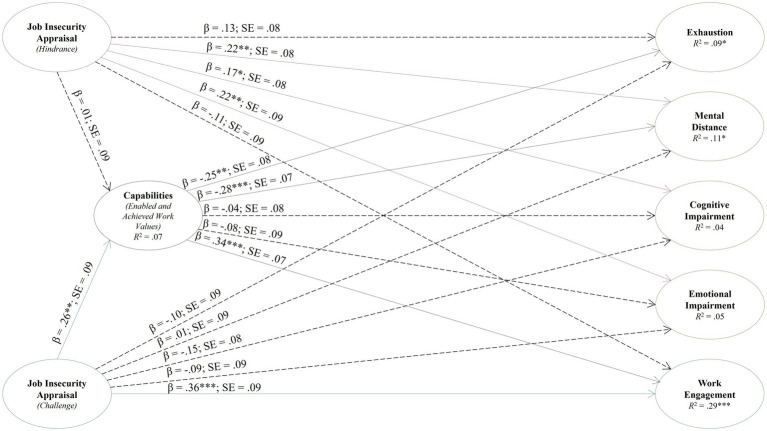
Structural model of job insecurity appraisal, capabilities, burnout, and work engagement. β = standardized estimates; SE = standard error; ***p < 0.001, **p < 0.01, *p < 0.05.

According to the results presented in Table 2 and illustrated in Figure 2, the perception of job insecurity as a hindrance does not have a statistically significant effect on the work capabilities of emergency nurses. Conversely, viewing job insecurity as a challenge has a statistically significant positive effect with a small effect size (*β* = 0.26). This potentially suggests that external factors such as environment context or individual differences might overshadow the potential impacts of perceiving job insecurity as a challenge. When emergency nurses view their job insecurity as a challenge, it may motivate them to actively seek methods to achieve valued work outcomes, thereby enhancing their functioning at work and preparing them for future opportunities.

Job insecurity perceived as a hindrance affects burnout, showing a statistically significant, small effect on three key dimensions: mental distance (*β* = 0.22), cognitive impairment (*β* = 0.17), and emotional impairment (*β* = 0.22). In contrast, viewing job insecurity as a challenge did not show a statistically significant effect on any burnout dimension among emergency nurses. This potentially suggests that the perception of job insecurity is crucial in determining its impact on well-being. Specifically, negative perceptions such as viewing job insecurity as a hindrance lead to more adverse outcomes, like burnout, while positive perceptions, such as seeing it as s challenge, may not significantly influence burnout.

Job insecurity perceived as a challenge was found to have a statistically significant, medium effect on work engagement (β = 0.36). Conversely, viewing job insecurity as a hindrance did not show a statistically significant effect on engagement among emergency nurses. This findings supports the notion that the perception of job insecurity significantly affects outcomes: a negative perception, such as viewing it as a hindrance, may lead to adverse outcomes like burnout, whereas a positive perception, viewing it as a challenge, is associated with beneficial outcomes such as increased work engagement.

Emergency nurses’ capabilities, defined as enabled and achieved work values, had a statistically significant small effect on their levels of exhaustion (*β* = −0.25) and mental distance (*β* = −0.28), but not on cognitive and emotional impairment. This discrepancy might be explained by the unique aspects of emergency nursing, where cognitive and emotional impairments are more directly tied to the intense emotional demands and cognitive overload inherent in the role (Adriaenssens et al., 2012; Caulfield et al., 2022; Surendran et al., 2024). Furthermore, these capabilities demonstrated a statistically significant impact on work engagement, showing a medium effect (β = 0.34). This indicates that higher levels of capabilities enhance work engagement among emergency nurses. This finding is particularly valuable as it provides empirical evidence that emergency nurses’ capabilities significantly impacts their work functioning, contributing to reducing burnout symptoms while promoting engagement.

As seen in Figure 2, emergency nurses’ work capabilities explained 7% of the variance [*R*^2^ = 0.07 (SE = 0.04; *p* = 0.090)]. Although the *p*-value was above the recommended value of 0.05, a pragmatic approach was followed as it was only off by 0.04. Also, this might be due to only the challenge appraisal toward job insecurity had a significant effect on emergency nurses’ capabilities. For burnout, exhaustion explained 4% [*R*^2^ = 0.09 (SE = 0.04; *p* = 0.040)] and mental distance 11% [*R*^2^ = 0.11 (SE = 0.05; *p* = 0.012)]. The emotional and cognitive impairment constructs showed insignificant *p*-values. This might be due to only the hindrance appraisal to job insecurity showing a significant effect on the impairment components of burnout among the sample of emergency nurses. Work engagement explained the most variance, 29% [*R*^2^ = 0.29 (SE = 0.07; *p* = 0.000)].

### 5.4 Latent profile analysis

Emergency nurses’ burnout and engagement profiles were investigated using factor scores saved from the measurement model. Table 3 reports the results of the five profiles computed.

**Table 3:** Comparisons of different burnout and engagement profile analysis models.

Profiles	AIC	BIC	aBIC	Entropy	LMR LR test *p*-value	aLMR LR test *p*-value	BLRT *p*-value	Smallest membership
One	2294.39	2327.58	2295.89	–	–	–	–	–
Two	1869.16	1922.25	1871.56	0.86	< 0.001	< 0.001	< 0.001	47%
Three	1702.59	1775.59	1705.89	0.86	0.179	0.186	< 0.001	24%
**Four**	**1619.48**	**1712.38**	**1623.67**	**0.89**	0.013*	0.015*	**< 0.001**	**12%**
Five	1596.56	1709.38	1601.65	0.90	0.427	0.435	< 0.001	2%

AIC, Akaike information criterion; BIC, Bayesian information criterion; aBIC, adjusted Bayesian information criterion; LMR LR, Lo–Mendell–Rubin test; aLMR LR, adjusted Lo–Mendell–Rubin test; BLRT, bootstrapped likelihood ratio test; *p < 0.05. Bold values refer to the selected model solution (i.e., four latent profiles).

As seen in Table 3, a four-profile solution provided superior fit indices compared to the other four profiles. Two profiles fitted the data better than one profile: ΔAIC = −425.23; ΔBIC = −405.33; ΔaBIC = −424.33; LMR LR (*p* < 0.001); aLMR (*p* < 0.001); BLRT (*p* < 0.001). Three profiles fitted the data better than two: ΔAIC = −166.57; ΔBIC = −146.66; ΔaBIC = −165.67; Δentropy = 0.00; LMR LR (*p* = 0.179); aLMR (*p* = 0.186); BLRT (*p* < 0.001). Four profiles fitted the data better than three: ΔAIC = −83.11; ΔBIC = −63.21; ΔaBIC = −82.22; Δentropy = 0.03; LMR LR (*p* = 0.013); aLMR (*p* = 0.015); BLRT (*p* < 0.001). While the AIC (Δ–22.92), BIC (Δ–3.00), aBIC (Δ–22.02), and entropy (Δ–0.01) values were better for five profiles compared to four profiles, the LMR LR and aLMR LR were non-significant compared to the significant *p*-values for the four-profile solution. Also, the smallest profile membership was 2%, which is not an appropriate group size. Subsequently, the four-profile solution was considered the best fit for the data.

**Figure 3:**
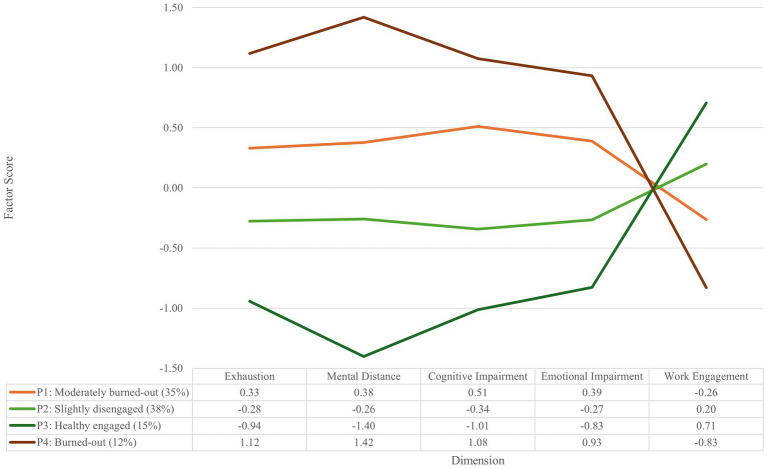
Emergency nurses’ latent burnout and engagement profiles.

Figure 3 illustrates the four latent burnout and engagement emergency nurse profiles identified. The four identified profiles (or subpopulations) comprised the following member allocations: Profile 1 had 71 (34.80%) members assigned to it, Profile 2 had 77 (37.74%), Profile 3 had 31 (15.20%), and Profile 4 had 25 (12.26%), indicating an acceptable proportion of member classification. The average latent profile probabilities were 0.93, 0.92, 0.97, and 0.95 for Profiles 1, 2, 3, and 4, respectively (Wang and Wang, 2020).

The profiles illustrated in Figure 3 can be interpreted as follows (adapted from Bekker et al., 2024): Profile 1: *moderately burned-out*. Emergency nurses who formed part of this profile reported slightly below-average work engagement and above-average burnout symptoms across all four burnout dimensions. Profile 2: *slightly disengaged*. Emergency nurses assigned to this group had slightly above-average work engagement and slightly below-average burnout symptoms across the four burnout dimensions compared to the other groups. Profile 3: *healthy engaged*. Emergency nurses assigned to this profile reported the highest level of work engagement and the lowest burnout symptoms across all the dimensions compared to the other emergency nurses, especially regarding mental distance. Profile 4: *burned-out*. Emergency nurses in this profile had the highest level of work disengagement while showing the highest burnout symptoms across all four of the burnout dimensions, especially regarding mental distance.

**Figure 4:**
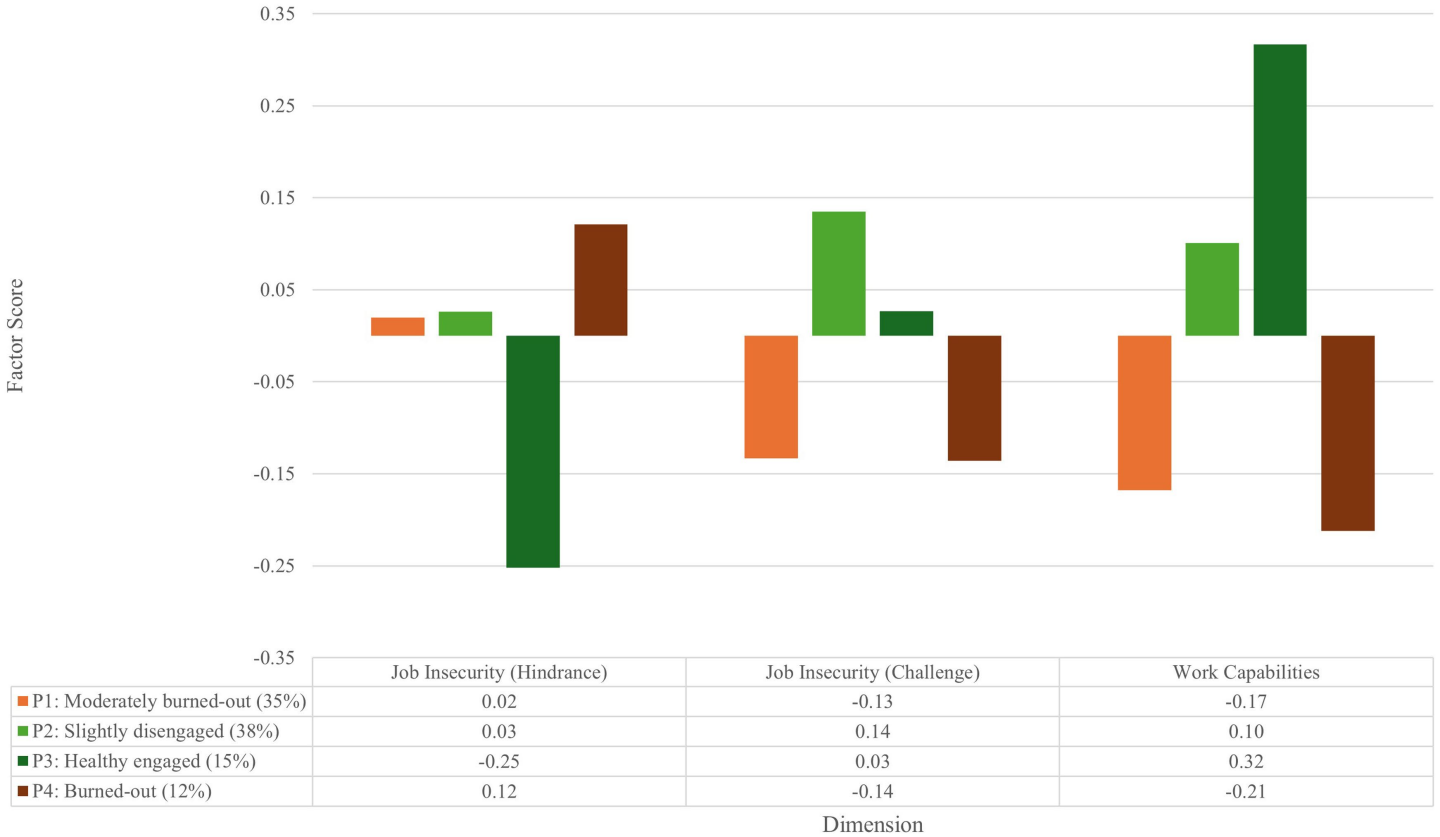
Burnout and engagement profiles, job insecurity appraisal, and capabilities of emergency nurses.

Figure 4 provides a visual representation of the identified latent emergency nurse burnout and engagement profiles based on their reported job insecurity appraisals and capabilities.

As seen in Figure 4, emergency nurses who were healthy engaged, reported the lowest hindrance job insecurity and the highest work capabilities compared to the other profiles (or subpopulations). Burned-out emergency nurses reported the highest job insecurity hindrance appraisal and the lowest work capabilities. The moderately burned-out and burned-out profiles did not experience job insecurity as a challenge and had noticeably lower work capabilities than those forming part of the slightly disengaged and healthy engaged profiles. Therefore, it is evident that those who experienced job insecurity as a hindrance and had low work capabilities reported low work engagement and at least mild burnout symptoms. Consequently, interventions aimed at addressing emergency nurses’ job insecurity appraisal, or at least viewing it as a challenge, could contribute to their level of work engagement and lower their burnout symptoms. Furthermore, interventions assisting emergency nurses in realizing work capabilities would substantially contribute to improving their work engagement and decreasing their burnout symptoms.

Table 4 presents the differences among emergency nurses’ latent burnout and engagement profiles in relation to their job insecurity appraisals (as either a hindrance or challenge) and their capabilities.

**Table 4:** Comparisons of different burnout and engagement profiles with job insecurity appraisal (hindrance and challenge) and capabilities.

Variable	*χ* ^2^	*p*	Profile	Mean *λ*	*SE*	Profile comparison	*χ* ^2^	*p*
Job insecurity (hindrance)	3.41	0.332	1	0.02	0.08	1 vs. 2	0.00	0.963
			2	0.03	0.10	1 vs. 3	2.19	0.139
			3	−0.25	0.17	1 vs. 4	0.47	0.492
			4	0.12	0.12	2 vs. 3	1.99	0.158
						2 vs. 4	0.39	0.535
						3 vs. 4	3.36	0.067
Job insecurity (challenge)	5.40	0.145	1	−0.13	0.08	1 vs. 2	4.21	0.040*
			2	0.14	0.10	1 vs. 3	0.86	0.355
			3	0.03	0.16	1 vs. 4	0.00	0.983
			4	−0.14	0.12	2 vs. 3	0.34	0.562
						2 vs. 4	3.23	0.072
						3 vs. 4	0.711	0.399
Work capabilities	20.81	<0.001	1	−0.17	0.08	1 vs. 2	5.54	0.019*
			2	0.10	0.08	1 vs. 3	15.55	<0.001***
			3	0.32	0.10	1 vs. 4	0.09	0.769
			4	−0.21	0.12	2 vs. 3	2.91	0.088
						2 vs. 4	4.82	0.028*
						3 vs. 4	11.73	0.001**

χ^2^ = chi-square; SE = standard error; Profile 1 = moderately burned-out; Profile 2 = slightly disengaged; Profile 3 = healthy engaged; Profile 4 = burned-out; ***p < 0.001, **p < 0.01, *p < 0.05.

As shown in Table 4, Profiles 1 (*moderately burned-out*) and 2 (*slightly disengaged*) differed significantly in their tendency to appraise job insecurity as a challenge. Additionally, Profiles 1 and 4 (*burned-out*) differed significantly from Profiles 2 and 3 (*healthy engaged*) in terms of their reported capabilities.

These findings suggest that emergency nurses’ placement within specific burnout and engagement profiles is impacted by their appraisal of job insecurity. Specifically, appraising job insecurity as a challenge (rather than a hindrance) and reporting higher levels of capabilities were associated with more favorable profiles, characterized by lower burnout symptoms and higher engagement at work.

## 6 Discussion

This study investigated how emergency nurses’ work capabilities and appraisals of job insecurity (as either a challenge or hindrance) relate to burnout and engagement, using the Sustainable Employability (SE) model (Van der Klink et al., 2016) as the guiding framework. It also aimed to identify distinct burnout and engagement profiles and determine how these profiles differ in terms of job insecurity appraisal and work capabilities. The findings offer theoretical, methodological, and practical contributions to the growing body of work on sustainable employability, specifically in high-demand and complex work environments.

The results revealed that emergency nurses’ capabilities, conceptualized as the real opportunities to pursue and realize personally valued work outcomes, were significantly associated with their levels of burnout and engagement. Specifically, higher capabilities were related to lower levels of exhaustion and mental distance, and to higher engagement. These findings support the SE model’s core proposition that when individuals have the freedom and support to pursue what they value at work, they tend to function better (Van der Klink et al., 2016; Fleuren et al., 2020; Gürbüz et al., 2024). This provides partial support for Hypothesis 1, which proposed that capabilities would be negatively associated with burnout (H_1a_) and positively with engagement (H_1b_).

However, capabilities were not significantly associated with the cognitive and emotional impairment dimensions of burnout. This distinction suggests that while capabilities may foster energy and commitment, they may be less effective in buffering against burnout symptoms related to cognitive processing and emotional regulation. These more severe symptoms may be shaped by other contextual stressors such as cumulative trauma exposure, persistent moral distress, or chronic psychological strain, challenges that are particularly salient in emergency care settings (Adriaenssens et al., 2011; García-Izquierdo and Ríos-Rísquez, 2012; Yang et al., 2024). This finding underscores the importance of distinguishing between motivational and regulatory dimensions of burnout and suggests that interventions aimed at fostering sustainable employability, especially among emergency nurses, may need to address both domains to be fully effective.

In addition to the role of capabilities, the findings demonstrated that emergency nurses’ job insecurity appraisals significantly affected their functioning at work. Emergency nurses who viewed job insecurity as a challenge reported higher levels of capabilities and engagement, while those who saw it as a hindrance reported higher levels of mental distance, cognitive impairment, and emotional impairment. These findings provide partial support for Hypotheses 2 and 3. Specifically, appraising job insecurity as a hindrance was associated with higher burnout symptoms (H_2b_), while appraising it as a challenge was positively associated with capabilities (H_3a_) and engagement (H_3c_).

These results align with prior research highlighting that job insecurity’s effects are not determined solely by its presence but by how it is interpreted (Charkhabi, 2019; Stynen et al., 2015). Emergency nurses who appraise insecurity as a challenge may be more likely to respond proactively; seeking support, leveraging available resources, or focusing on professional development. In contrast, those who view it as a hindrance may be more prone to disengagement and emotional strain. This also supports intervention strategies aimed at helping nurses reframe job insecurity through mentoring, coaching, or reflective dialog, particularly in emergency contexts characterized by uncertainty, pressure, and limited autonomy (Afaya et al., 2021; Power et al., 2022).

The latent profile analysis (LPA) revealed four distinct subpopulations based on burnout and engagement: burned-out (12%), moderately burned-out (35%), slightly disengaged (38%), and healthy engaged (15%). The burned-out group emerged as the most at-risk, reporting the highest levels of all burnout dimensions and the lowest levels of engagement. Although they comprised only 12% of the sample, the severity of their symptoms suggests an urgent need for targeted intervention. This group also reported the lowest capabilities and tended to appraise job insecurity as a hindrance, reinforcing the role of both contextual constraints and psychological appraisals in shaping vulnerability. Without support, individuals in this profile are likely to experience serious consequences such as emotional exhaustion, impaired cognitive and emotional functioning, poor patient care, and intention to leave the profession.

In contrast, the healthy engaged group exhibited the lowest burnout scores (especially mental distance) and the highest engagement. This group also reported the highest capabilities and the strongest tendency to appraise job insecurity as a challenge. Although small in size, their profile offers encouraging evidence that thriving is possible, even in demanding emergency care contexts.

The majority of emergency nurses were distributed across the middle two profiles: moderately burned-out and slightly disengaged. These groups reflect a more precarious position; neither engaged nor burned out. With the right organizational support, these individuals could potentially shift toward greater engagement, but without it, they may deteriorate further. Capability-building and appraisal-focused interventions may therefore be instrumental in shifting these trajectories toward sustainable employability.

The final set of analyses investigated whether burnout and engagement profiles differed in terms of job insecurity appraisal and capabilities. The Bolck-Croon-Hagenaars (BCH) analysis revealed significant differences across the profiles. Emergency nurses in the moderately burned-out group appraised job insecurity as significantly less of a challenge than those in the slightly disengaged group. Additionally, the moderately burned-out and burned-out profiles reported significantly lower capabilities than the slightly disengaged and healthy engaged profiles. These differences partially supported Hypothesis 5, which proposed that burnout and engagement profiles would differ significantly in terms of job insecurity appraisal and work capabilities.

These findings carry both theoretical and practical implications. Theoretically, the study supports and extends the SE model by demonstrating how contextual appraisals and individual capabilities interact to shape complex patterns of burnout and engagement. Practically, the findings suggest that healthcare organizations should prioritize interventions that enable employees to realize valued work outcomes and foster constructive interpretations of job insecurity. Capability-enhancing practices (such as providing autonomy, opportunities for growth, and meaningful contributions) combined with initiatives that support adaptive appraisal (such as mentoring, reflective practice, and leadership support), could significantly improve emergency nurses’ functioning at work.

While situated within the South African context, where resource constraints and inequality between the public and private healthcare sectors shape work experiences, these findings also have relevance beyond emergency nursing. Other high-risk, high-demand professions (such as social work, policing, and academic work) may also benefit from integrative approaches that simultaneously address capabilities, context, and appraisal, and warrants further investigation.

Together, the findings provide insight into the psychosocial mechanisms that support sustainable employability in complex and precarious work environments, and lay the foundation for further research and intervention tailored to diverse occupational settings.

## 7 Study contributions

This study makes several contributions to the literature on sustainable employability, emergency nursing, and occupational well-being. Theoretically, it affirms the Sustainable Employability (SE; Van der Klink et al., 2016) model by showing that work capabilities, defined as enabled and achieved work values, are essential for reducing burnout symptoms (particularly exhaustion and mental distance) and enhancing engagement. The findings strengthen the argument that sustainable functioning at work depends not only on resources, but also on individuals’ ability to pursue and realize personally valued outcomes.

The study further extends existing research by highlighting job insecurity appraisal as a critical contextual factor. Emergency nurses who interpreted job insecurity as a challenge reported greater capabilities and engagement, while hindrance appraisals were linked to increased burnout symptoms. These results reinforce the importance of appraisal processes in shaping sustainable employability outcomes.

From a methodological perspective, the study contributes by applying a person-centered approach to identify four distinct burnout and engagement profiles: moderately burned-out, slightly disengaged, healthy engaged, and burned-out. These findings demonstrate that burnout and engagement are not binary opposites but can co-exist in meaningful combinations. The use of latent profile analysis offers a nuanced understanding of occupational well-being and helps uncover hidden subgroups within high-stress professions.

Finally, the study shows that capabilities and job insecurity appraisals differ significantly across these profiles. Emergency nurses in the more engaged profiles reported greater capabilities and challenge appraisals, whereas those in more burned-out profiles showed the opposite pattern. This provides further support for the SE model’s assertion that sustainable employability is shaped by both contextual conditions and individuals’ perceived opportunities.

## 8 Limitations and recommendations for future research

While this study provides valuable insights into the interplay between job insecurity appraisals, work capabilities, burnout, and engagement among emergency nurses, framed within the Sustainable Employability (SE) model, several limitations should be acknowledged.

To begin with, the cross-sectional design restricts the ability to make causal inferences and limits understanding of the stability of the identified burnout and engagement profiles over time. Longitudinal research is needed to track transitions between profiles and to clarify the directionality of relationships, particularly the potential bidirectional influence between job insecurity appraisals and capabilities.

In addition, the study focused on emergency nurses in a single South African province, predominantly within the private healthcare sector. Although a range of hospitals were included, the findings may not generalize to public sector contexts or other provinces. Broader geographic and institutional representation in future studies would help to uncover more comprehensive and context-sensitive patterns.

Moreover, although the sample of 204 emergency nurses was sufficient for the analytic strategy employed, a larger and more diverse sample might reveal additional or more nuanced burnout and engagement profiles. Increasing sample heterogeneity in future research could also support deeper investigation of the systemic and organizational factors that shape sustainable employability.

Finally, the use of a convenience sampling strategy limits the generalizability of the findings. While emergency nurses were intentionally targeted due to their relevance to the research aims, participation was based on hospital willingness and accessibility, which likely introduced selection bias. The predominance of private hospitals and the limited representation of the public sector (due to challenges in securing permissions) suggests that the perspectives of emergency nurses working in public settings, who may face different constraints, are underrepresented.

Taken together, these limitations highlight the need for future longitudinal, cross-sectoral, and large-scale studies. Such research would not only strengthen the evidence base for the Sustainable Employability model but also provide critical insights to inform policy development, workforce planning, and targeted support strategies for emergency nurses across diverse healthcare settings.

## 9 Conclusion

This study examined how emergency nurses’ work capabilities and job insecurity appraisals affect burnout and engagement, using the Sustainable Employability model as a guiding framework. The findings demonstrate that greater capabilities are associated with reduced exhaustion and mental distance, and with higher engagement, affirming their relevance as a resource for sustainable functioning in high-demand healthcare environments. Moreover, how emergency nurses interpret job insecurity plays a critical role. Challenge appraisals were linked to greater capabilities and engagement, while hindrance appraisals were associated with elevated burnout symptoms, particularly mental distance, cognitive, and emotional impairment.

The identification of four distinct burnout and engagement profiles further highlights the variability in emergency nurses’ experiences. Those in the healthy engaged profile reported the highest capabilities and challenge-oriented appraisals, while those in the burned-out profile exhibited the opposite pattern. These findings reinforce the importance of person-centered approaches in understanding and addressing occupational well-being.

As pressures within emergency care settings persist, fostering work environments that enable the pursuit of valued outcomes and promote adaptive appraisals of job insecurity is essential. Doing so will support not only the well-being and sustainable employability of emergency nurses but also the resilience of the broader healthcare system.

## Data Availability

The raw data supporting the conclusions of this article will be made available by the author on request, without undue reservation.
